# Extraction of Pregabalin in Urine Samples Using a Sulfonated Poly(ether ether ketone) Membrane

**DOI:** 10.1155/2021/3439242

**Published:** 2021-05-31

**Authors:** Chanbasha Basheer

**Affiliations:** ^1^Department of Chemistry, King Fahd University of Petroleum and Minerals, Dhahran 31261, Saudi Arabia; ^2^Membranes and Water Security, Interdisciplinary Research Center, KFUPM, Dhahran 31261, Saudi Arabia

## Abstract

In this work, a simple polymer-assisted microextraction technique was developed to determine pregabalin (an anticonvulsant drug) in the urine sample. A sulfonated poly(ether ether ketone) membrane was used as a sorbent for pregabalin extraction, and the extraction performance was compared with that of the conventional polydimethylsiloxane membrane. The extraction device is free moving and tumbles continuously throughout the stirred sample solution during extraction to enhance the extraction efficiency. The electrostatic interactions between the sulfonic-acid-functionalized polymeric membrane and the amine group in the pregabalin molecule facilitate higher preconcentration factor at a shorter extraction time. Optimizing conditions of the extraction method were investigated to obtain higher extraction efficiency. The developed method exhibited good linearity in the range of 0.05 to 2 *µ*g/mL with a correlation of determination (*r*^2^) 0.9998, acceptable limits of detection, limits of quantification, and preconcentration factor of 105-fold. The within-day and between-day precisions of pregabalin were lower than 7% relative standard deviations. Pregabalin was extracted from urine samples with recoveries of >92%, and no significant matrix effects were observed.

## 1. Introduction

**Figure 1:**
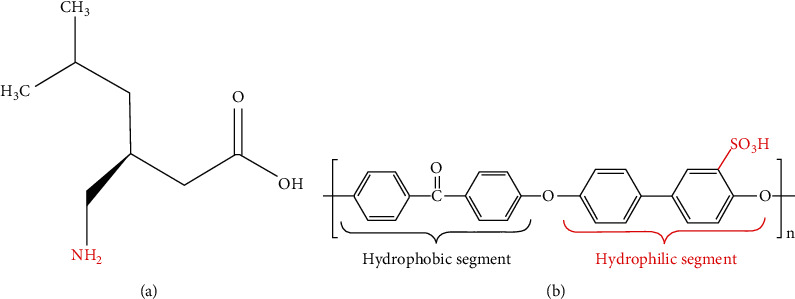
Chemical structure of (a) pregabalin and (b) SPEE.

Pregabalin is used as adjunctive therapy for partial seizures with or without secondary generalization in adults. Pregabalin (S-3-(aminomethyl)-5-methyl hexanoic acid) is a structural derivative of the inhibitory neurotransmitter aminobutyric acid. In recent research, pregabalin has been confirmed for adjunctive treatment of partial seizures to treat neuropathic pain from postherpetic neuralgia and diabetic neuropathy in adults in both the United States and Europe [1, 2]. The chemical structure of pregabalin is shown in Figure 1(a). Pregabalin is available under the trade name Lyrica (Pfizer, New York, NY) for use for treating epilepsy, diabetic neuropathy pain, postherpetic neuralgia [3], effective at fibromyalgia [4], and spinal cord injury [5].

The precise mode of action of pregabalin has not been fully elucidated. However, it does interact with the same binding site and has a similar pharmacologic profile as gabapentine (1-[aminomethyl] cyclohexane acetic acid) [6, 7]. Pregabalin is minimally metabolized and primarily excreted in urine as unchanged drugs, and studies in healthy volunteers indicate oral bioavailability to be approximately 90% [8]. It is available in 25 mg, 50 mg, 75 mg, 150 mg, and 300 mg capsules, and this variation allows it to be easier to prescribe when the medication is being introduced.

In clinic, dizziness and somnolence are the most frequently reported adverse events, with dizziness experienced by 29% of pregabalin-treated patients compared with 9% with placebo and somnolence experienced by 22% of pregabalin-treated patients. Decreased concentration, increased appetite and weight gain, dry mouth, and vomiting are other side effects [9].

On top of that, increasing slow-wave sleep in healthy volunteers is another side effect correlated with the sleep's restorative aspect.

A relatively uncomplicated and more cost-effective method such as spectrophotometry or spectrofluorometry is the basic requirement for routine analysis of the drug in bulk powder and pharmaceutical preparations, particularly in research laboratories and the pharmaceutical industry. Literature review revealed that conventional direct analysis or liquid-liquid extraction methods were used to analyze pregabalin in tablets or biological matrices. After extraction, analyses were performed by gas chromatography-mass spectrophotometry (GC-MS), high-performance liquid chromatography (HPLC)-MS-MS [10, 11], HPLC [11–16], and fluorometry [17] without any derivatization.

However, to improve the direct analysis's sensitivity, various derivatization reagents were used; for example, cyclodextrins were added before capillary electrophoresis and nuclear magnetic resonance analysis [18]. Other chromogenic reagents such as 2,3-dichloro-5,6-dicyano-1,4-benzoquinone (DDQ) and 7,7,8,8-tetracyanoquinodimethane (TCNQ) or 2,4-dinitrofluorobenzene and 2,3,5,6-tetrachloro-1,4-benzoquinone and ninhydrin were used for UV/visible spectrophotometry or spectrofluorometry analysis [2, 19–21].

The derivatization method requires additional tedious derivatization steps, which further dilute the analytes' concentrations [11, 22] and are challenging for trace-level quantitation. Unfortunately, the excess derivatization reagents often result in resolution and detection problems such as difficulty to separate a trace target derivative in the presence of a significant excess of an unreacted reagent. The excessive reagent might interfere with the chromatographic separation process [23].

It is well known that, in sorptive processes, higher extraction efficiencies are obtained when the analytes are in their nonionic form. Various sorptive extraction techniques using polydimethylsiloxane (PDMS) have been reported for the extraction drug samples in urine samples which include solid-phase microextraction [24] and stirrer bar sorptive microextraction (SBSE) [25, 26]. Recently, functionalized polymers were used to extract a wide range of organic compounds with high selectivity and enrichment [27]. The selective extraction mechanism of analytes was explained by various interactions, which includes chelation [28, 29], chiral recognition [30–32], ion-exchange through electrostatic interactions [33–35], creation of recognition sites for target analytes using molecularly imprinted polymers [36–38], and immunoaffinity through antibody-antigen interactions [39–42].

In this study, for the first time, we have developed polymer-assisted microextraction (PME) using a sulfonated poly(ether ether ketone) (SPEEK) functionalized polymer membrane as an adsorbent. After extraction, the extract was injected into HPLC (without any derivatization). The developed method required only small amounts of sample and solvent.

## 2. Materials

### 2.1. Chemicals

Sulfonated poly(ether ether ketone) (Fumatech, ion-exchange capacity = 1.6 mmole/g) and sodium hydroxide were purchased from Fluka (AG, Switzerland). Pregabalin (99.5%) was obtained from Symed Laboratories (AP, India). HPLC-grade organic solvents were purchased from Sigma-Aldrich (St. Louis, MO, USA). The polydimethylsiloxane (PDMS) polymer comprising the Slygard 184 silicone elastomer and a curing agent was purchased from Dow Corning Corporation (Midland, MI, USA). Ultrapure water was used for the preparation of all standard solutions and mobile phase. Stock standard solution of pregabalin was prepared by dissolving 50 mg of pregabalin into 25 mL distilled water. Standard solutions of pregabalin (2.0, 1.5, 1, 0.5, 0.1, and 0.05 *µ*g/mL) were prepared by subsequent dilution.

### 2.2. Urine Samples

Urine samples were collected from a healthy volunteer at the university campus. The samples were taken in amber glass bottles previously rinsed with methanol and ultrapure water, and the samples were stored in the dark at 4°C for a maximum of 48 hours. Before their analysis, urine samples were filtered using cellulose acetate membranes (0.45 *μ*m pore size). In order to test the accuracy of the method, the drug-free urine samples were spiked with a known amount of pregabalin standard.

## 3. Instrument

The Waters HPLC system was used in the study with a *µ*-Bondapak C_18_ column (3.9 × 300 mm) (USA). The flow rate was adjusted to 1 mL/min, and the injection volume used was 10 *µ*L with a retention time of 15 mins. A Waters 2996 Photodiode Array was used as the detector, and the wavelength of detection was 210 nm. The mobile phase was prepared by dissolving 1.2 g of monobasic potassium phosphate in 940 mL water. Then, the pH of the resultant solution was adjusted to 6.90 (by using 5N NaOH solution), then 60 mL of acetonitrile was added, and the solution was degassed.

## 4. Membrane Preparation

In this study, sulfonated poly(ether ether ketone) (SPEEK), with the chemical structure shown in Figure 1(b), is used as the acid-functionalized polymer to prepare the corresponding acid-functionalized membrane.

Dry SPEEK polymer was dissolved in dimethylacetamide (DMAc) solvent to form an approximately 10 wt.% solution. Next, the solution was poured into a glass plate, the solvent was slowly evaporated, and the resulted membrane was peeled off by immersing in deionized water for a few hours. The residual solvent was then removed by drying the obtained membrane in a vacuum oven. The dried membrane was conditioned by soaking in 0.5 M H_2_SO_4_, washed with deionized water, and finally, dried at 60°C for 3 hours.

**Figure 2:**
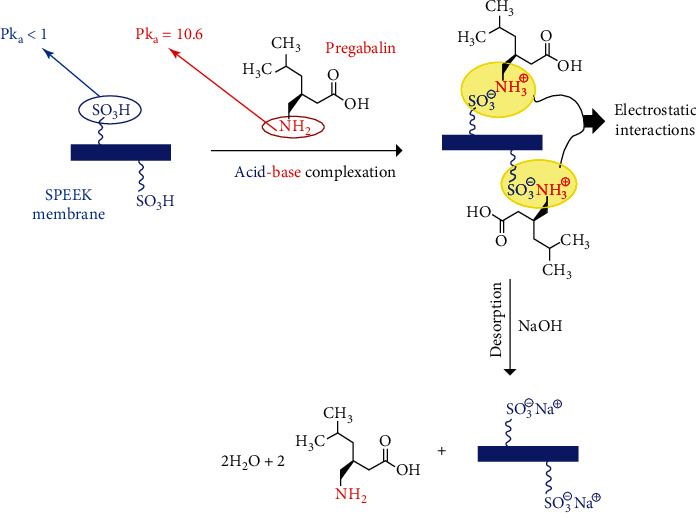
Illustration for the ion-exchange mechanism and the electrostatic interactions between the SPEEK membrane and pregabalin analyte.

The attached sulfonic acid functionality on the poly(ether ether ketone) backbone makes the polymer surface more hydrophilic. It provides robust, accessible acidic ion-exchange sites for possible interaction with analytes containing basic nitrogen functionality such as pregabalin, as illustrated in Figure 2. Moreover, the extraction performance of PME was compared for polymeric adsorbents using SPEEK and the commercial polydimethylsiloxane (PDMS) membrane.

## 5. Polymeric-Membrane-Assisted Microextraction (PME)

50 mg of the SPEEK membrane was placed into a 10 mL drug-free urine sample spiked with various pregabalin concentrations, and the sample was agitated with a stir bar for 2 min. Then, the polymeric sorbent was removed from the sample vial and wiped softly with tissue.

The pregabalin-extracted SPEEK membrane was desorbed in 500 *µ*L of 0.1 M NaOH solution via ultrasonication for 2 min. Finally, the membrane was removed from the HPLC autosampler vial, and 10 *µ*L of the extract was injected into HPLC. The SPEEK membrane was conditioned again with a large volume of NaOH solution for 10 min to check the carryover effect; no analyte was detected after the second desorption. This indicates that the membrane is suitable for subsequent extraction. To condition and regenerate the SPEEK membrane, the membrane was ultrasonicated with 50 mL of 0.5 M H_2_SO_4_ for 5 min and used for further extraction.

## 6. Results and Discussion

### 6.1. Optimization of PME

The extraction principle of PME is based on ion-pair partitioning of pregabalin with the SPEEK membrane. The ion-exchange extraction mechanism of SPEEK provides a convenient pathway to the simultaneous sampling, sample preparation, and preconcentration of pregabalin. The extraction parameters affecting PME, such as extraction time, desorption time, desorption solvent, and sample pH, were optimized. These are the factors that were considered to play an essential role in determining extraction efficiency.

### 6.2. Selection of Polymeric Sorbent

**Figure 3:**
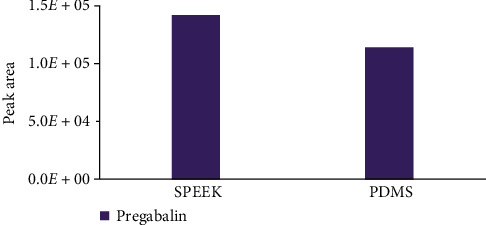
Comparison of SPEEK and PDMS as a sorbent. Extractions were performed at identical conditions.

The extraction performance of PDMS and SPEEK membranes were tested at identical conditions (2 min extraction time, 0.1 M NaOH desorption solvent with 2 min desorption time). Compared with PDMS, SPEEK showed improved extraction ability for the pregabalin analyte, as shown in Figure 3. The enhanced extraction ability of the SPEEK membrane could be attributed to the hydrophilicity of the acid-functionalized polymer as well as the presence of electrostatic interactions between the grafted sulfonic acid moiety on the polymer backbone (Pk_a_ < 1) and the amine group of the pregabalin analyte (Pk_a_ = 10.6), as illustrated in Figure 2. The significant Pk_a_ difference allows for fast and effective ionic crosslinking and successful electrostatic interaction between the SPEEK and the–NH_2_ functionality on the pregabalin molecules. Hence, the SPEEK membrane was further selected as the functionalized extraction sorbent for method development.

### 6.3. Extraction Time

**Figure 4:**
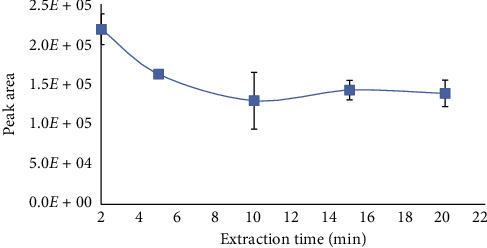
Extraction profile of pregabalin using PME.

The extraction was monitored over the range of 2 to 20 min.  Figure 4 shows the behavior of pregabalin under different extraction periods. The HPLC signals decreased to an extraction time of 2 min, and extraction efficiency remains approximately constant after 10 minutes. Faster mass transfer of the analyte was achieved due to the functionality of the SPEEK membrane. Thus, 2 min extraction time was selected for further analysis.

### 6.4. Desorption Solvent

After extraction, the SPEEK-containing analyte was desorbed with an organic solvent via ultrasonication. Two important factors should be considered before selecting a suitable solvent for desorption of the analyte from SPEEK: (i) the polymer should be insoluble in the desorbing solvent, and (ii) analytes must be soluble in the solvent.

In the present case, the SPEEK membrane with ion-exchange capacity (IEC = 1.6) is soluble in polar solvents such as methanol, ethanol, and acetonitrile. However, since the adsorption mechanism is based on electrostatic interactions and ion pair formation, the desorption solvent shall be able to break the complexed ion pairs by neutralization of the charged polymeric functionalities (−SO_3_H/−CO_2_H) as illustrated in Figure 2. Based on the abovementioned discussion, a 0.1 M NaOH solution was selected as the desorption solvent for pregabalin elution from the SPEEK membrane. Finally, NaOH extract was injected directly into the HPLC system.

### 6.5. Desorption Time

The effects of the desorption period (ultrasonication time using a 0.1 M NaOH solution as a desorption solvent) on the pregabalin extraction were studied. According to the literature, the desorption time required using a solvent is usually no longer than 20 min, and mechanisms to facilitate the desorption process may be ultrasonication or agitation [43]. On this basis, desorption time between 2 and 20 min was investigated. Since the extraction process is based on an ion-exchange mechanism, the analyte was quickly desorbed in a shorter time (2 min). After each extraction and desorption, the SPEEK membrane was rinsed with acetone for 2 min to avoid any analyte carryover problem.

### 6.6. Effect of Sample pH

The effect of sample pH on the pregabalin's extraction efficiency was evaluated in the pH range of 2–12. The highest response was obtained at the lowest pH 2. The obtained highest response in the acidic environment could be attributed to the efficient ion pair electrostatic interactions between the SPEEK membrane and pregabalin analyte. In other words, when the medium becomes less acidic, the ion-exchange capacity of SPEEK polymer decreases, which means that the number of free acidic protons (H^+^) decreases; thus, the number of free acidic sites available for interaction with the amine groups of pregabalin became less, which led to a slight reduction in the instrument response. Although the maximum obtained response at pH = 2 is about one order of magnitude higher than the lowest response at pH > 6, this observation supports the mechanism of electrostatic interaction and ion pair formation in the studied PME system. In comparison, when the medium became alkaline (pH > 7), the sulfonic acid groups neutralized and become negatively charged, which makes them inaccessible for the formation of ion pairs with basic (-NH_2_) functional groups of the pregabalin analyte. Regardless, the obtained constant response in the alkaline pH range could be attributed to the improved surface hydrophilicity (Figure 1(b)) of the polymeric membrane due to the grafted polar functional groups (−SO_3_^−^). Hence, in the alkaline medium, the adsorption-desorption mechanism could be suggested due to both the pregabalin analyte and SPEEK membrane's hydrophilic nature.

### 6.7. Effect of Salt Addition

The addition of salt to the sample solution may decrease the polar analyte's solubility and increase the partition coefficient, and this process is called the salting-out effect. To assess the salting-out effect on the extraction of pregabalin from urine samples, various NaCl concentrations were added to urine samples in the range of 1–20% wt/vol. The addition of salt to the urine samples does not significantly improve the extraction (data not shown).

To illustrate, when NaCl is added in high concentration to the analyzed urine samples, H^+^ protons of the sulfonic acid groups in SPEEK polymer are exchanged with Na^+^ protons. As a result, HCl is liberated.

Hence, the sulfonic acid groups became neutralized by sodium counterions and unable to form ion pairs with basic functionalities (−NH_2_). This result provided evidence for the critical role of the enhanced surface hydrophilicity of the SPEEK membrane, which facilitates the adsorption-desorption of hydrophilic analytes with polar functionalities such as pregabalin. Therefore, no salt was added to the urine samples in the reported experiments.

### 6.8. Quantitative Analysis

Based on the experiments discussed above, the optimal PME conditions were: as follows 50 mg of SPEEK was used, sample pH of 2, and an extraction time of 2 min. After the extraction, the analytes were desorbed using 500 *µ*L of 0.1 M NaOH by ultrasonication for 2 min. 10 *µ*L of the extract was injected into the HPLC system. To evaluate the PME technique, repeatability, linearity, and detection limits under the optimal extraction conditions were investigated. The repeatability in peak areas was studied for three replicate experiments (3 separate pieces of SPEEK). The relative standard deviations (RSDs) were lower than 7%. Extraction of pregabalin exhibited good linearity over the concentration range 0.05–2 *μ*g/mL under optimum conditions. Coefficients of correlation (*r*^2^) better than 0.999 were obtained. LOD and LOQ were calculated as 3.3 *σ*/s and 10 *σ*/s, respectively, as per International Conference on Harmonization (ICH) definitions, where *σ* is the mean, standard deviation of replicate determination values under the same conditions as the sample analysis in the absence of the analyte (blank determination), and *s* is the sensitivity, namely the slope of the calibration graphs. LOD values for pregabalin were 0.03 ng/mL and LOQ 0.09 ng/mL, respectively. The PME method obtained 105-fold enrichment.

**Figure 5:**
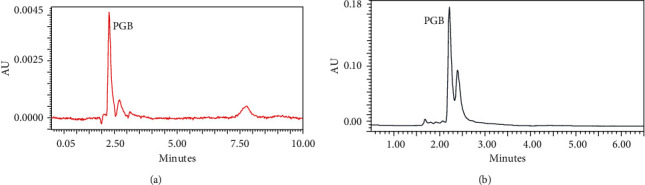
HPLC chromatogram of pregabalin (a) before extraction at 5 *µ*L spiked sample and (b) after SPEEK extraction of drug-free urine sample spiked at 0.1 *µ*g/mL.

The PME method was compared with already reported methods in the literature and summarized in Table 1. The results demonstrate the applicability of the method for routine trace-level analysis of pregabalin from a urine sample. The PME method's recovery was investigated with a healthy person's urine samples, and the samples were spiked with the known concentration of pregabalin, 0.5 and 1.5 *µ*g/mL, respectively. The extraction recoveries were calculated and reported in Table 2, and recoveries varied between 90 and 92%, respectively, which indicates that SPEEK polymer can successfully extract the pregabalin from urine samples more effectively. These results demonstrate the absence of significant matrix effects on the efficiency of PME.  Figure 5 shows the HPLC chromatograms of (a) the pregabalin standard sample with a concentration of 5 *µ*g/mL and (b) the SPEEK extract drug-free urine sample spiked with 0.1 *µ*g/mL pregabalin.

## 7. Conclusions

In this work, a polymer-assisted microextraction coupled with HPLC is developed to rapidly determine the amount of pregabalin in urine samples. We reported the potential use of the acid-functionalized SPEEK as an ion-pair low-cost sorbent for selective sample enrichment of pregabalin from urine samples. The PME device was allowed to tumble freely in the sample solution to enhance the extraction efficiency. A small amount of low-cost sorbent, reusability of the sorbent up to 50 times without loss of analytes after consecutive extractions, and the extreme simplicity of the procedure are the main advantages of this technique. Also, the method permits the determination of analytes at low concentrations showing good performance over the commercial PDMS membrane. [46]

## Figures and Tables

**Table 1:** Quantitative parameter of PME.

	Spiked	Detected	Recovery	Spiked	Detected	Recovery (%)
	Concentration (mg/L) (*n* = 3)			Concentration (mg/L) (*n* = 3)		
Pregabalin	0.5	0.45	90.1	1.5	1.39	92.3

**Table 2:** Comparison of the methods reported in the literature for pregabalin.

Method	SV (ml)	E T	LOD	%RSD	RR	Ref
LLE (manual)	50	10 min	4.8 ng/mL	0.17	99.5–101	[44]
LLE (ultrasonication)	50	∼15 min	66.9 ng/mL	0.068–0.167	97.12–98.86	[13]
LLE (manual)	15	3 min	1 ng/mL	11.4%.	69.8–72%	[45]
PME	20	2 min	0.03 ng/mL	6.8%	90–92	Current study

SV: sample volume (mL). ET: extraction time (min). LOD: limit of detection (ng/mL). RSD: relative standard deviation (%). RR: relative recovery (%).

## Data Availability

The data used to support the findings of this study are included within the article as figures and tables.
